# Nano-enabled approaches in virology: emerging strategies for rapid detection, antiviral therapy, and biomimetic innovation

**DOI:** 10.3389/fbioe.2026.1904030

**Published:** 2026-10-20

**Authors:** Deepali Rustagi, Kunal, Rohit Kumar, Pankaj Kumar, Atin Kumar, Rajeev, Komal Verma, Debasis Mitra, Mahendru Kumar Gautam, Rohit Kumar Singh, Caleb Ayomide Babatunde

**Affiliations:** 1 Department of Life Sciences, School of Allied Health Sciences, SGT University, Gurugram, India; 2 Faculty of Agricultural Sciences, GLA University, Mathura, Uttar Pradesh, India; 3 Department of Entomology, Sardar Vallabh Bhai Patel University of Agriculture and Technology, Meerut, India; 4 School of Agriculture, Uttaranchal University, Dehradun, Uttarakhand, India; 5 Department of Agronomy, School of Agriculture, Lovely Professional University, Phagwara, Punjab, India; 6 School of Pharmacy, Babu Banarasi Das University, Lucknow, India; 7 Department of Microbiology, Graphic Era (Deemed to be University), Dehradun, Uttarakhand, India; 8 School of Agricultural Sciences, Babu Banarasi Das University, Lucknow, Uttar Pradesh, India; 9 School of Agricultural Sciences, Babu Banarasi Das University, Lucknow, India; 10 Pedology and Land Use Research Group, Department of Soil Resources and Environmental Management, Ekiti State University, Ado Ekiti, Nigeria

**Keywords:** biosensors, nanobiotechnology, nanoparticles, viral diagnostics, viral-mediated nanoparticle synthesis, virus

## Abstract

Global health continues to be impacted by viral diseases, and this poses a need for timely diagnosis, potent antiviral treatment, and flexible prevention. To tackle these problems, nanotechnology has become an incredibly flexible tool for viral detection, therapeutic delivery, vaccination and biomimetic material design. Recent developments in the field of nano-enabled virology will be reviewed and will focus on the use of nanomaterials in the development of biosensors and diagnostic platforms to make virus detection more sensitive, specific, faster and more portable. It also explores the antiviral activity of metallic, polymeric, lipid-based, carbon-based and 2D nanomaterials, including MXenes, their ability to directly inactivate viruses and targeted delivery of antiviral agents and nucleic acids, etc. Special focus is placed on lipid nanoparticles as carriers for RNA therapeutics and mRNA vaccines, and novel vaccine strategies based on peptides, proteins, virus-like particles, and DNA. Virus-templated and virus-inspired nanomaterials are also included in the review and are considered as biomimetic platforms for the controlled fabrication of functional nanostructures. Special emphasis is placed on MXenes, which are currently being explored for the detection and application against viruses, due to their tunable surface chemistry and potential future applications, and challenges associated with stability, toxicity, and translation are highlighted. Finally, ongoing translational issues such as safety, toxicity, production, regulatory issues and the development of experimental platforms to clinical application are addressed.

## 1 Introduction

Viral diseases remain one of the most serious socioeconomic and health threats to humanity, as demonstrated by the regular outbreaks of pathogens such as the Ebola virus, human immunodeficiency virus (HIV), and SARS-CoV-2 (Leligdowicz et al., 2016; Morens et al., 2004; Zhou et al., 2020). The high prevalence of viral mutations and adaptation impedes detection and control efforts and, consequently, results in high morbidity, mortality, and economic instability (Dawood, 2020; Yuwen et al., 2023).

The history of virology is based on the original discovery of infectious agents and involves the study of the structure, genomics, and replication of viruses, which form the fundamental principles for developing antiviral therapy (Mercer et al., 2010; Nasir et al., 2012). Nevertheless, despite significant advances in molecular virology, traditional diagnostic and treatment solutions do not always hold up when faster, more sensitive, and adaptable to new viral strains are required (Yuwen et al., 2023; Zhang et al., 2020).

Historically, classical techniques such as nucleic acid amplification, immunological testing (antigen/antibody), and viral culture represent the foundation of modern diagnostic testing for viral infections; however, these classic techniques have technical and practical limitations. For example, reverse transcriptase polymerase chain reaction (RT-PCR), loop-mediated isothermal amplification (LAMP), and enzyme-linked immunosorbent assay (ELISA) tests are considered very accurate because of their high sensitivity and specificity; however, they require special equipment, a controlled laboratory environment, and personnel with adequate training (Notomi et al., 2000; Deshpande et al., 2021; Smyrlaki et al., 2020). Although viral culture is still considered the most reliable method for identifying a virus, it requires too much time (days to weeks) and extensive labor for culture isolation and cannot be performed in the field (Steinhardt et al., 1913; Feng et al., 2021). Additionally, immunological detection systems may produce false-positive results due to technical errors resulting from the presence of lower quantities of virus in the sample, that is, low (substantial portions of the population can produce false-negative results) or high levels of viral load in the sample in question (Peng et al., 2022). Therefore, new, rapid, reliable, and scalable virus detection systems and methods that can produce accurate results must be developed as soon as possible (Huang et al., 2020; Yuwen et al., 2023).

Nanotechnology, which originated in the fields of science, engineering, and technology, has created new opportunities for manipulating materials and biological systems at the nanoscale. The International Organization for Standardization (ISO) defines nanotechnology as a science that involves the creation of materials with dimensions of less than 100 nm. Materials developed at the nanoscale tend to display distinct physical and chemical properties owing to their large surface areas, quantum phenomena, and programmable surface chemistries. (Ratan et al*.,* 2021; Soares et al*.,* 2018). These properties enable interactions between nanoparticles and viral and cellular particles, facilitating rapid detection and intervention (Zhou et al., 2021). Nanoscale studies combined with virology have led to the field of nanobiotechnology, in which the molecular understanding of viruses is integrated with the functional versatility of nanomaterials (Kargozar and Mozafari, 2018). A schematic representation of the different types of nanoparticles (NPs), highlighting their key structural features and functional characteristics relevant to biomedical applications, is presented in Figure 1.

**FIGURE 1:**
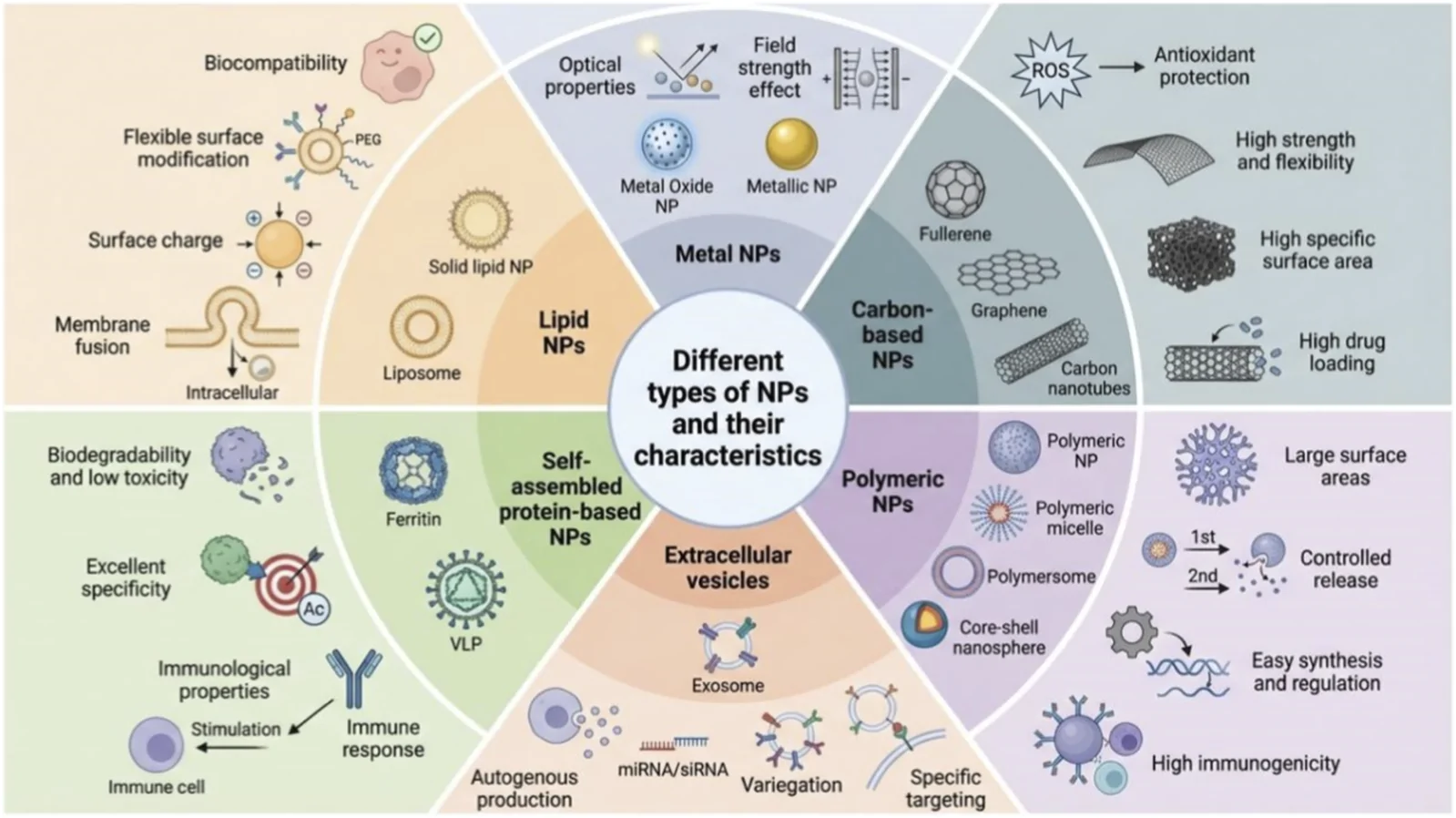
Schematic overview of different types of nanoparticles highlighting their key structural features and functional characteristics relevant to biomedical applications.

Nanotechnology-based detection systems have great potential for addressing these challenges. Nanoparticle-based biosensors, especially those that adopt gold and silver nanoclusters, quantum dots, or up-conversion nanoparticles, take advantage of the optical, electrical, and catalytic peculiarities of nanoparticles to enable high signal-to-noise ratios, photostability, and surface modifiability, making them excellent candidates for next-generation viral biosensing systems (Mokhtarzadeh et al., 2017; Xu et al., 2023). Fluorescence-based technologies utilizing metal nanoclusters or rare-earth-doped NPs have provided significant functionality in early diagnosis, pathogen tracking, and bioimaging (Xiao et al., 2021; Qian et al., 2022). All these nanotechnological advances combine a basic understanding of virology with real-time diagnostics.

In addition to their diagnostic capabilities, NPs exhibit direct antiviral activity. Silver (Ag), gold (Au), copper (Cu), and iron (Fe) oxide are metallic NPs with broad-spectrum anti-viral effects that can disrupt capsids, block the viral genome replication cycle, and avoid the host-virus interactions (Rai et al., 2014; Fujimori et al., 2012; Kumar et al., 2019; Tavakoli and Hashemzadeh, 2020). AgNPs specifically show the ability to counter a wide range of viruses, as demonstrated by their attachment to viral surface proteins and restriction of the entry or replication of viruses, such as HIV, influenza, and respiratory syncytial virus and others (Lara et al., 2010; Xiang et al., 2011; Ratan et al., 2021). Further improvements in selectivity and reduction in cytotoxicity via biomolecule functionalization enhance the therapeutic capability of these NPs (Jackman et al., 2016).

The application of nanotechnology in virology is inherently reciprocal. Viruses are known as natural biomimetic templates for nanomaterial synthesis, with their well-structured capsid architecture at the nanometer scale, which can self-assemble, making them likely scaffolds (Jain et al., 2021; Dhaka et al., 2023). Virus-mediated AgNPs are considered green and sustainable compared to traditional chemical methods because viral proteins or nucleic acids serve as reducing and capping agents (Ahmad et al., 2024; Telkapalliwar and Deharkar, 2025). The biocompatibility and structural homogeneity of plant viruses, such as the tobacco mosaic virus (TMV) and cowpea mosaic virus (CPMV), are among the leading topics in this research field (Jain et al., 2021; Ivanov et al., 2023).

Recent studies have studied various aspects of the nanotechnology–virology interface, though mainly in related application and material contexts. For instance, Goharshadi et al. (2022) have reviewed nanotechnology applications against viral infection, focusing on diagnostic sensors at the nanoscales, vaccine formulations, antiviral nanocoatings, nanocarriers and therapeutic applications. Ali et al. (2023) then performed a material-science based evaluation of antiviral nanomaterials, mainly concentrating on the direct antiviral activity of nanoparticles as well as on their application as carriers for antiviral therapeutic agents. In an complementary direction Zhang et al. (2018) reported plant viruses as biotemplates for the fabrication of nanomaterials, highlighting the structural uniformity, self-assembly and templating properties of the viral architectures. All these studies together demonstrate the wide range of possibilities of nanotechnology applications in the field of virology, covering in isolation the diagnostic, therapeutic and virus-templated aspects.

The present review extends these contributions by taking the virology/nanotechnology interface as a two-way and mechanistically connected interface. In particular, it focuses on three complementary aspects: (i) Nano-enabled viral detection: From fluorescent nanoparticles to metal nanoclusters, the upconversion nanoparticles to nanoscale biosensors; (ii) nanoparticle-mediated antiviral intervention at various stages of the viral life cycle; (iii) Viral architectures as biomimetic or biotemplating systems for the controlled fabrication of nanomaterials. Instead of considering these applications separately, we connect the structure and function of viruses to the physicochemical properties of nanomaterials, and explore the duality of these connections, namely, nanomaterials acting on viruses as well as viral structures acting as templates or design principles for the development of nanomaterials. In our knowledge this integrated analysis of viral detection, antiviral activity, and virus-templated nanofabrication in one mechanistic context has not been given the same level of attention in the review literature. This view allows for a single concept of nanotechnology as a tool to combat viral infections and as a platform based on viral self-assembly and structure.

## 2 Methodology

This narrative review was prepared after critically examining the role of nanoscale engineering in virology, with an emphasis on viral biosensing, antiviral nanomaterials, and virus-templated nanoparticle synthesis. Peer-reviewed research articles, reviews, and seminal studies published primarily between 2010 and 2026 were collected from PubMed, ScienceDirect, and Google Scholar databases.

Relevant literature was identified using a combination of keywords, such as nanoparticle-based viral detection, fluorescent nanoparticles, metal nanoclusters, upconversion nanoparticles, two dimensional nanoparticles, MXenes, Raman spectroscopy for viral diagnostics, antiviral properties of nanoparticles, silver nanoparticles, virus-templated nanoparticle synthesis, biomimetic nanomaterials, and nanobiotechnology in virology. Only studies that reported clear mechanistic insights, experimental validation, or analytical performance metrics were included to ensure scientific rigor.

## 3 Viruses and virology

Viral diseases are one of the greatest dangers to humans because they have triggered several pandemics in the past. Viruses are suggested to have co-evolved with the precursors of modern eukaryotic cells and have a multiplicity of ancient cellular lineages (Nasir et al., 2012). The threat posed by viral infectious diseases is severe on a global scale (Yuwen et al., 2023). The significance of the prevention and control of viral diseases was also emphasized by the COVID-19 pandemic, which is caused by SARS-CoV-2 (Ke et al., 2020; Yao et al., 2020). They have taken away vast masses of people to different viruses, causing colossal losses in terms of the economy and lasting destruction to civilization.

### 3.1 Viruses and their capsid geometry

Viruses exhibit a wide range of sizes and shapes, measuring 20–900 nm, and can infect bacteria, fungi, plants, and mammals (Passi et al., 2015). To enable host cell invasion and protect viral genetic material, the virion is surrounded by an outer protein coat, referred to as the capsid (Mercer et al., 2010). Most viruses have an extra envelope that covers the capsid and provides additional functionality and defense (Rey and Lok, 2018). This envelope is usually formed by the plasma membrane of the host cell and a lipid bilayer (Zhou et al., 2021). Viral capsids are of three broad types: complex, helical, and icosahedral (Johnson and Chiu, 2000). The protein subunits of helical viruses form a helix around an axis, and positively charged nucleic acids are bound to negatively charged amino acids to fix the genome. Viruses such as HIV use almost spherical, hollow, icosahedral capsids to carry their genomes. Many viruses, especially bacteriophages, have acquired capsids that combine the features of helical and icosahedral structures (Zhou et al., 2021).

### 3.2 Transmission, early detection and virus cycle

Corstjens et al. (2000) stated that the virus could be transmitted horizontally or vertically, that is, person-to-person and mother-to-child. Some of these transmission routes are sexual contact, whereas others are fecal-oral through contaminated food or water. Direct contact, salivary exchange, coughing, and sneezing can also transmit viruses. In addition, hepatitis viruses or chronic HIV infection may advance to malignancy or acquired immunodeficiency (Ratan et al., 2021). They are highly transmissible, and their control measures have negative effects on the overall health of the population (Morens et al., 2004).

Viruses damage cells, tissues, and organs and cause severe infectious diseases by hijacking and replicating within host cells (Zhang et al., 2020). SARS-CoV-2, smallpox, bird influenza, acquired immunodeficiency syndrome, Ebola hemorrhagic fever, and many other deadly viral diseases are all virus-borne, as are the common cold, influenza, and chickenpox (Anesten et al., 2016; Leligdowicz et al., 2016; Xu et al., 2020; Zhou et al., 2020).

Viruses have also developed mechanisms that enable them to attack a vast array of host organisms with ease. This is because of the virus’s high epidemiological fitness; a single virus particle or virion can reproduce and initiate a productive infection cycle in a new host. Viruses are obligate intracellular parasites; therefore, they must infect host cells to replicate themselves. As a result, almost every virus follows a comparable order of entry, replication, and release throughout its life cycle, regardless of its structural properties (Zhou et al., 2021).

Viral replication is rapid, and there are high levels of mutation during transmission, making early diagnosis critical for controlling the disease and managing outbreaks. Early detection and treatment of virus transmission are effective measures for containment efforts (Wolters et al., 2020). Therefore, it is essential to develop efficient and rapid viral detection methods.

## 4 Role of nanotechnology in virology

Over the past few years, breakthroughs in nanotechnology have advanced virology, particularly in creating discrete nanoscale systems. At the 100 nm level, nanomaterials have been developed to produce high-performance biomolecular systems capable of selectively targeting specific cells, viruses, bacteria, and fungi (Zhou et al., 2021). This high surface-to-volume ratio, along with the high concentration of surface atoms in the NPs, confers specific physical and chemical properties (plasmonic resonance, fluorescence enhancement, and catalysis), which have led to their use as antiviral agents (Shabatina et al., 2023). These distinct characteristics enable early disease diagnosis, disease prevention, tissue-selective therapy, individualized nanomedicine, and the production of safer and more effective drugs (Soares et al., 2018). This, in turn, has resulted in an abundance of nano-based therapeutics to increase targeted delivery and therapeutic efficacy against viral pathogens (Lembo et al., 2018). Since the outbreak of the COVID-19 pandemic, interest in nanotechnology in academia and industry has increased significantly (Aranda et al., 2020; Jones et al., 2020; Talebian et al., 2020).

Nanomaterials have enabled major advancements and discoveries in bioanalytical research because of their unique physicochemical properties (Tang et al., 2021). The high specificity and sensitivity, flexible sensing modalities, label-free and real-time detection in biosensors, and other characteristics of nanostructure-based biosensors have generated significant interest in nanotechnology and biosensor research (Mokhtarzadeh et al., 2017). Recent studies have focused on the synthesis of nanotechnology and fluorescent biosensing systems. Biosensors based on fluorescence offer a number of native benefits that encourage early diagnosis and enhanced healthcare outcomes: (1) extremely low detection limits and increased sensitivity; (2) quick and visual readout to facilitate timely diagnosis; and (3) cost-efficient detection (Xu et al., 2023).

New nanotechnological products can address these limitations and have the potential to develop broad-spectrum nanotherapeutic platforms for viral infections (Fang et al., 2018; Kargozar and Mozafari, 2018; Kim et al., 2010). Some nanomaterials exhibit inherent antiviral toxicity, enabling direct viral inhibition (Vazquez-Muñoz et al., 2017). Further progress in the functionalization and structural modification of nanoparticles has enabled the selective binding and elimination of pathogens (Jackman et al., 2016).

The scale of research and development in nanotechnology for virus treatment has created a host of opportunities that outweigh those offered by traditional antiviral methods. In addition to improving existing treatments, nanomaterials provide alternative antiviral pathways and can eliminate or suppress viruses through distinct mechanisms (Zhou et al., 2021). Figure 2 illustrates the role of nanotechnology in virology.

**FIGURE 2:**
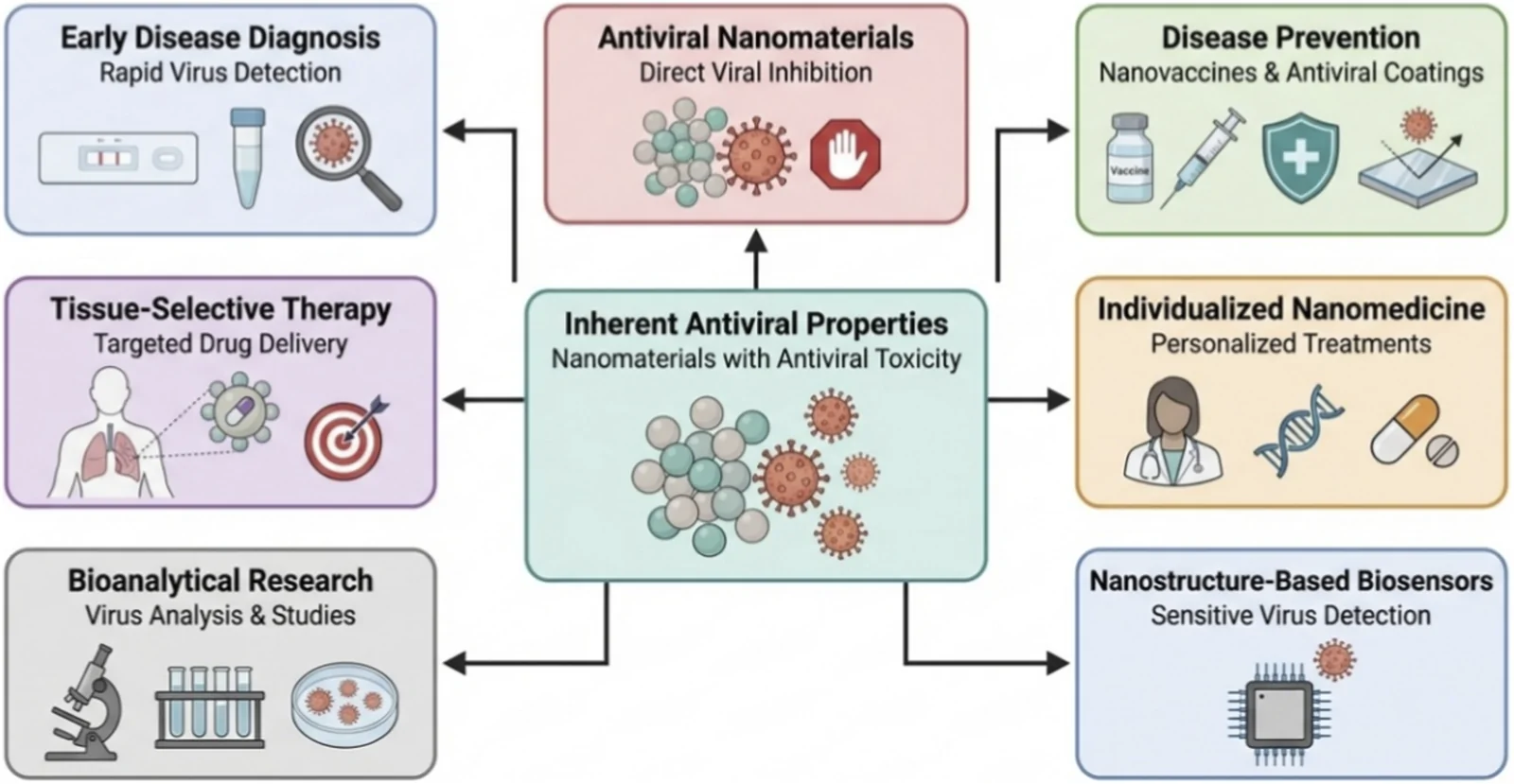
Illustration of the role of nanotechnology in virology.

### 4.1 Traditional virus detection techniques

High sensitivity and the ability to quickly identify viral pathogens are imperative for removing the source of infection and preventing disease transmission. Current viral diagnostics largely rely on nucleic acid and protein detection (Amanat et al., 2020; Chen et al., 2015; Ye et al., 2022). Table 1 shows the principles, advantages, limitations, and applications of various traditional viral detection techniques.

**TABLE 1:** Overview of traditional virus detection techniques, summarizing their principles, commonly used methods, advantages, limitations, and applications.

Detection technique	Principle	Common methods	Advantages	Limitations	Typical applications	Citation
Culture-Based Techniques	Isolation and propagation of viruses in host cells or culture media	Viral culture, Cell culture assays	Gold standard; enables study of viral infectivity, pathogenesis, and drug testing	Time-consuming; labour-intensive; requires biosafety facilities and expertise	Viral characterization, vaccine development, antiviral drug testing	Steinhardt et al. (1913); Feng et al. (2021); Ziegler et al. (2020); Xu et al. (2023); Yuwen et al. (2023)
Immunological-Based Techniques	Detection based on antigen–antibody interaction generated by immune response	ELISA, Immunochromatographic assays, Rapid diagnostic tests	Fast detection; high specificity; suitable for field testing	Reduced sensitivity in early infection or low viral load; false positives possible; prior antigen knowledge required	Point-of-care diagnostics, epidemiological surveillance	Yuwen et al. (2023); Peng et al. (2022)
Nucleic Acid-Based Techniques	Detection and amplification of viral DNA or RNA sequences using sequence-specific primers	PCR, RT-PCR, qPCR, Isothermal amplification (LAMP, RPA)	High sensitivity and specificity; early infection detection; rapid identification	Expensive instrumentation; skilled personnel required; primer mismatch due to viral mutation may reduce detection efficiency	Clinical diagnostics, outbreak surveillance, molecular epidemiology	Amanat et al. (2020); Chen et al. (2015); Ye et al. (2022); Yuwen et al. (2023)
Protein-Based Techniques	Detection of viral antigens or host antibodies produced against viral proteins	ELISA, Serum neutralization assay, Immunocolloidal gold test, Indirect immunofluorescence	Rapid, cost-effective, suitable for large sample screening	Lower sensitivity compared to molecular methods; cross-reactivity may occur	Serological testing, routine diagnostics, immune response monitoring	Deshpande et al. (2021); Jackson and Balfour (1988)

#### 4.1.1 Culture-based technique

First applied to the growth of the cowpox virus, the isolation-and-culture technique has undergone extensive development and is currently considered the gold standard for identifying and testing viruses (Steinhardt et al., 1913). This method has contributed to the disciplines of viral pathogenesis, vaccine research, and treatment research in an essential way (Feng et al., 2021; Ziegler et al., 2020). Nevertheless, clinical teams increasingly require the urgent detection of pathology to achieve optimal therapeutic outcomes, and culture-based methods may not deliver these results in a timely manner in many cases (Yuwen et al., 2023). Virus culture is both labor- and time-intensive, which means that it requires skilled personnel and exact procedures; hence, it is not very useful in rapid diagnostics. Therefore, alternative detection methods with high sensitivity and accuracy should be developed (Xu et al., 2023).

#### 4.1.2 Immunological-based techniques

Immunological tests are important for disease detection, as they involve the generation of specialized antibodies by the human immune system. Standard immunological tests, such as enzyme-linked immunosorbent assay (ELISA) and colloidal gold immunochromatography, offer specificity and speed, making them suitable for effective diagnostic and surveillance assays to detect and monitor infectious diseases in the near future. Immunological detection has enabled the design of efficient therapeutic and diagnostic methods for infectious diseases (Yuwen et al., 2023).

Although immunological methods are essential for identifying viruses, they have some limitations (Peng et al., 2022), including the inability to recognize small amounts of viral antigens or antibodies in samples with low viral loads or in early infections. In addition, false-positive outcomes may occur due to the presence of non-target viruses or disruptive chemicals that can affect the diagnostic results. In addition, the specific antigens or antibodies related to the target virus must be known in advance. Another disadvantage is the lack of information on viral subtypes or mutations, which can reduce the detection fidelity.

#### 4.1.3 Nucleic acid-based technique

The nucleic acid-based system entails the recognition of viral DNA or RNA, and it is sensitive and specific; however, the system is laborious and expensive (Yuwen et al., 2023). Nucleic acid amplification is commonly used in molecular biology to identify and amplify specific DNA or RNA sequences within biological samples. These methods fall into the categories of polymerase chain reaction (PCR) and isothermal amplification.

Moreover, viral genetic variation may lead to a mismatch between primers and the target sequence, reducing amplification efficiency or resulting in no detection of the target. Addressing these limitations is a central challenge in the development of virus detectors (Yuwen et al., 2023).

#### 4.1.4 Protein-based technique

Protein-based detection methods include antigen and antibody detection. Antigen detection focuses on viral surface antigens, whereas host-derived antibodies target viral antigens. Protein immunophenotyping is an efficient tool for quick and affordable detection; however, it can be less sensitive and specific than other methods. Methods to detect proteins include serum neutralization, ELISA (Deshpande et al., 2021), immunocolloidal gold test, and indirect immunofluorescence (Jackson and Balfour, 1988).

### 4.2 Nanoparticle-based virus detection techniques

Nanoparticle-based virus detection has rapidly advanced as a promising alternative to traditional diagnostic methods, offering rapid, sensitive, and portable solutions for identifying a wide range of viruses in clinical, agricultural, food safety, and environmental contexts (Lu et al., 2011; Sarza et al., 2025; Zhang et al., 2022). These technologies leverage the unique optical, electrical, and magnetic properties of nanoparticles, such as gold, silver, magnetic particles, and quantum dots, to enhance the signal generation and target capture. Applications span from point-of-care (POC) diagnostics for human pathogens, such as SARS-CoV-2 and hepatitis viruses, to monitoring foodborne and plant viruses across the farm-to-fork chain (Draz and Shafiee, 2018; Sarza et al., 2025; Zhang et al., 2022). While many platforms remain at the proof-of-concept stage, recent studies have demonstrated that nanoparticle-based biosensors can achieve high sensitivity (down to single-digit copies/mL), rapid turnaround times (hours to minutes), and compatibility with smartphone or AI-assisted readouts (Draz et al., 2020). However, challenges such as sample matrix effects, manufacturing scalability, nanomaterial stability, and regulatory hurdles must be addressed before widespread adoption (Xu et al., 2023). Overall, nanoparticle-based virus detection is poised to play a critical role in future outbreaks and routine surveillance.

#### 4.2.1 Organic dye-doped fluorescent nanoparticles

Organic fluorescent dyes contain conjugated aromatic or heterocyclic structures that enable fluorescence emissions. However, free dyes often exhibit poor photostability, low quantum yield, and aggregation-induced quenching in aqueous environments, limiting their utility in viral diagnostics (Jiao et al., 2020; Xu et al., 2023). The encapsulation of dyes within nanoparticles significantly enhances the fluorescence intensity, photostability, and resistance to photobleaching, making organic dye-doped fluorescent nanoparticles (ODFNs) highly effective signal reporters.

In virus detection, ODFNs function as signal-amplification probes by conjugating nanoparticle surfaces with virus-specific biomolecules, such as antibodies, aptamers, or nucleic acid probes. Upon binding to viral antigens or genomes, the nanoparticles emit strong fluorescence signals proportional to the viral concentration. Since each nanoparticle can encapsulate thousands of dye molecules, ODFNs provide substantial signal amplification, enabling the highly sensitive detection of low-abundance viruses (Luo et al., 2020).

Silica- and polymer-based dye-doped nanoparticles are widely used because of their excellent biocompatibility and ease of surface functionalization. For example, Luo et al. (2020) developed Ru (bpy)_3_^2+^-doped silica nanoparticles (RuSi NPs) for detecting the H9N2 avian influenza virus. These nanoparticles exhibited approximately 1000-fold higher electrochemiluminescence signals than free dye molecules and enabled virus detection with an extremely low detection limit of 140 fg mL^-1^ when combined with immunomagnetic separation.

Similarly, dye-doped fluorescent nanoparticles functionalized with antibodies against the SARS-CoV-2 spike protein have been used in fluorescence immunoassays for the rapid and highly sensitive detection of viral antigens (Tao et al., 2023). These nanoparticles significantly improved the detection sensitivity and enabled early stage diagnosis by enhancing the signal stability and amplification. In another example, dye-doped polymer nanoparticles conjugated with HIV-specific DNA probes have been used in fluorescence-based nucleic acid detection assays (Childress et al., 2012). These nanoparticles enabled the highly sensitive detection of HIV RNA by improving the fluorescence signal intensity and reducing background interference, demonstrating their potential for early viral diagnosis and monitoring.

Overall, ODFNs play a crucial role in viral diagnostics by enhancing the fluorescence signal output, improving the detection sensitivity, and enabling selective virus recognition. Dye-doped silica and polymer-based fluorescent nanoparticles have demonstrated femtogram-level detection sensitivity in viral immunoassays and biosensing platforms, highlighting their importance in sensitive and reliable virus detection (Table 2).

**TABLE 2:** Various nanoparticles used in viral detection.

Nanoparticle system	Virus target	Dye/label used	Polymer/matrix	Detection method/notes	Citation
Fluorescent magnetic multifunctional nanospheres	H9N2, H1N1, H7N9 Avian Influenza	Not specified (multicolour encoded)	Magnetic polymer nanospheres	Digital single virus immunoassay, multiplexed, limit of detection (LOD) 0.02 pg/mL	Wu et al., 2019)
Functional silica nanospheres (RuSi NPs)	H9N2 Avian Influenza	Ru (bpy)_3_^2+^ (tris(bipyridine)ruthenium (II))	Silica	Electrochemiluminescence (ECL) immunosensor, LOD 14 fg/mL	(Luo et al., 2020)
Magnetic T4 phage SPR probe	Influenza A Virus (IAV)	T4 phage labelled with scFv and biotin	Streptavidin-modified magnetic nanoparticle	Visual counting under dark field microscopy, LOD 1 PFU/μL	(He L et al., 2025)
Fluorescent nanodiamonds (FNDs)	Avian Influenza Virus	Nanodiamonds with NV-centers	-	Spin-enhanced lateral flow immunoassay (SELFIA), LOD 0.87 ng/mL	(Angela et al., 2025)
Polymeric nanoparticles (PNPs)	Influenza A Virus	Self-quenched fluorophores	Amphiphilic copolymers	Fluorescence turn-on via viral fusion peptide insertion	(Park et al., 2021)
Fe_3_O_4_@Ag magnetic SERS nanotags	Influenza A H1N1, Adenovirus	Raman dye molecules	Fe_3_O_4_@Ag core-shell	SERS-based lateral flow immunoassay, LOD 50 pfu/mL (H1N1)	(Wang et al., 2019)
Magnetic core-shell silica nanoparticles	Nucleic Acids (general, including viral)	4-hydroxy coumarin, 7-hydroxy coumarin fluorescein	Fe_2_O_3_@SiO_2_	Enhanced nucleic acid visualization, LOD improved over ethidium bromide	(Ali et al., 2025)
Upconversion nanoparticle-based lateral flow immunoassay (UCNP-LFIA)	SARS-CoV-2	NaYF_4_:Yb^3+^,Er^3+^ UCNPs conjugated with anti-N protein antibodies	Lanthanide-doped NaYF_4_ nanocrystals	NIR-excited fluorescence LFIA; LOD 3.59 pg/mL; ∼100-fold higher sensitivity than AuNP LFIA	(Ding et al., 2023)
UCNP-based quantum optical immunoassay	SARS-CoV-2	UCNPs functionalized with receptor binding domain (RBD) proteins	Rare-earth doped UCNP nanocrystal	Neutralizing antibody detection using UCNP fluorescence imaging; sensitivity ≈4 ng/mL	(Rajil et al., 2021)
Microfluidic chip-assisted upconversion luminescence biosensing platform	Respiratory viruses (including Influenza and SARS-CoV-2 model systems)	NaYF_4_:Yb^3+^,Er^3+^ upconversion nanoparticles	Lanthanide-doped UCNPs integrated into microfluidic chip	NIR-excited upconversion luminescence biosensor for portable point-of-care virus diagnostics with low autofluorescence; LOD 1.12 pg mL^-1^	(Liu et al., 2024)
Quantum-dot-functionalized Ti3C2 MXene	Influenza A virus; SARS-CoV-2	Quantum dots	Ti3C2 MXene	Dual-functional immunochromatographic assay; colorimetric and fluorescent detection; LOD 2.4 pg/mL for influenza A and 6.2 pg/mL for SARS-CoV-2; assay time ∼20 min	Liu et al. (2023)
HFGNs/MXene/PPy nanocomposite	Rotavirus	Methylene blue	Gold nanostructure/MXene/polypyrrole	Electrochemical genosensor using an ssDNA probe; LOD ∼0.8 aM; high selectivity and stability	Ketabi et al. (2023)
MXene quantum dots (MQDs)	Viral targets including influenza A, rotavirus, hepatitis B, Zika virus and SARS-CoV-2	Target-specific antibodies/nucleic-acid probes	MXene quantum dots	Used in electrochemical, photoelectrochemical and photothermal viral sensing platforms; high surface-to-volume ratio and surface functional groups support probe immobilization	Majeed et al. (2026)

#### 4.2.2 Metal nanoclusters

Metal nanoclusters (MNCs) are composed of metal atoms and ligands, and their size can be compared to the electronic Fermi wavelength. The creation of nanomaterials is rapidly growing owing to their small size (2 nm or less) (Zheng et al., 2021), good photophysical characteristics, high biocompatibility, and large specific surface area (Kang and Zhu, 2019; Xiao et al., 2021). However, in recent years, novel methods for the synthesis of MNCs, particularly Au, Ag, and Cu nanoclusters (NCs), have been developed (Qian et al., 2022). MNCs have tunable fluorescence and are easier to synthesize than standard organic dyes, making them more appealing for virus detection of viruses (Xu et al., 2023).

Metal NCs are efficient probes for fluorescence and chromaticity signals because of their high fluorescence and catalytic performance (Xiao et al., 2021). Similarly, interactions between MNCs and analytes can alter the metal core, ligand shell, or microenvironment, thereby changing their functional properties. Accordingly, MNCs are used as identification and signal conversion elements and are highly selective and sensitive for virus detection. Metal NCs, including Au, Ag, and Cu, have become a standard for production and have delivered excellent results in biomarkers, biosensing, bioimaging, and bioassays (Qian et al., 2022). Representative applications of MNCs in viral sensing, including detection limits and assay formats, are summarized in Table 2.

Based on a Cas12a-based electrochemiluminescence (ECL) biosensor, L-methionine-stabilized AuNCs were used to detect HPV, with a Limit of Detection (LOD) of 0.48 pM and a 70 min analysis time (Dai et al., 2019; Liu et al., 2021). AuNC biosensors based on impedance could detect HBV with an LOD of 0.1M and RSD of 1.39% (Shariati, 2021); also, HIV could be detected by AuNC graphene hybrids at 30 a.m. (Wang et al., 2015). AgNC templates and DNAs allowed the detection of the H5N1 virus at a LOD of 500 pM of fluorescence using AgNCs (Zhang et al., 2020), and the detection of HPV16 using AgNCs and a color shift at a LOD of 2 nM (Wang et al., 2015). CuNCs have also been employed for visual detection of the SARS-CoV-2 Delta variant using rationally designed AT-rich primers (Du et al., 2022). Separately, Tao et al. (2022) conducted a head-to-head comparison of DNA-templated AuNCs, AgNCs, and CuNCs within the same CRISPR-Cas12a fluorescence assay for HBV DNA detection. The CuNC-based assay showed a detection limit of 0.54 pM and a response time of 25 min, compared with 78.24 pM and 2 h for AgNCs and 162.41 pM and 12 h for AuNCs, respectively. Because these values were obtained within the same study and assay framework, they provide a direct comparative assessment under those experimental conditions; however, they should not be generalized across different viral targets or assay configurations.

#### 4.2.3 Up-conversion nanoparticles

Upconversion nanoparticles (UCNPs) are widely used in viral diagnostics because of their ability to convert near-infrared (NIR) excitation into visible fluorescence emission through anti-Stokes luminescence. This property enables highly sensitive virus detection with minimal background autofluorescence, as biological samples exhibit negligible fluorescence under NIR excitation (Chen et al., 2018; Xu et al., 2023).

In virus detection, UCNPs primarily function as fluorescent signal reporters by conjugating their surfaces with virus-specific antibodies, aptamers, and nucleic acid probes. Upon binding to viral antigens or viral genetic material, UCNPs emit strong fluorescence signals under NIR excitation, enabling highly sensitive and selective detection of the target. Their high photostability and resistance to photobleaching allow for reliable detection, even at low viral concentrations.

UCNP-based fluorescence immunoassays have been successfully used to detect influenza viruses. For example, antibody-functionalized UCNPs have been used to detect influenza virus antigens with significantly greater sensitivity than that of conventional fluorescent probes. Similarly, UCNP-based fluorescence resonance energy transfer systems have been developed for the detection of viral nucleic acids, including HIV RNA, enabling the highly sensitive and specific identification of viral genomes.

UCNPs have also been used in lateral flow immunoassays for SARS-CoV-2 detection, in which UCNPs conjugated to antibodies bind to viral spike or nucleocapsid proteins and generate detectable fluorescence. These UCNP-based assays demonstrated improved sensitivity and quantitative detection capabilities compared to traditional gold nanoparticle-based assays, making them suitable for rapid, early-stage viral diagnosis.

Overall, UCNPs provide highly sensitive, stable, and low-background fluorescence signals, making them valuable tools for viral immunoassays, nucleic acid detection, and biosensing platforms. Their application in detecting the influenza virus, HIV, and SARS-CoV-2 demonstrates their significant potential for improving the sensitivity and reliability of modern viral diagnostics.

#### 4.2.4 MXenes for viral detection

MXenes are hydrophilic, have a high electrical conductivity, and have a high surface area, as well as an abundance of surface functional groups such as–OH, –O and–F. The properties of these allow for biomolecule immobilization and render MXenes as excellent transducer materials for antibodies, nucleic-acid probes and other recognition elements (Zamiri et al., 2023).

There are electrochemical, field-effect transistor, fluorescent and surface-plasmon based MXene-based biosensors. Ti_3_C_2_Tx is the most widely studied MXene while Nb-, V- and Mo-based MXenes have been investigated. They have both conductivity and surface chemistry that are suitable for the modification and immobilization of virus-specific recognition molecules. Reports of applications for the detection of HIV-1, HPV, influenza A, hepatitis B and SARS-CoV-2 have been reported (Zamiri et al., 2023).

In recent years, MXene based electrochemical platforms for detecting the influenza A, rotavirus, hepatitis B, Zika virus, and SARS-CoV-2 have been proven. MXenes have been employed as sensing layers or as part of nanocomposites to enhance the surface properties of the electrodes and aid in biomolecule immobilization. Ti_3_C_2_-based immunosensing has been reported in the context of H1N1 influenza and MXene-based platforms have been investigated for SARS-CoV-2 nucleic-acid detection (Parihar et al., 2024).

Quantum-dot-functionalized Ti_3_C_2_ MXene has also been used for simultaneous detection of influenza A virus and SARS-CoV-2. The dual-functional immunochromatographic assay gave colorimetric and ‘fluorescent’ results within about 20 min with limit of detection (LOD) of 2.4 pg/mL for influenza A and 6.2 pg/mL for SARS-CoV-2 (Liu et al., 2023). These are expanded by MXene quantum dots (MQDs) which offer a high surface-to-volume ratio and surface groups that allow antibody and nucleic-acid probes to be attached. They have been studied for electrochemical, photoelectrochemical and photothermal viral sensing platforms (Majeed et al., 2026).

The analytical signals can also be improved by adding MXenes. Ketabi et al. (2023) prepared a hierarchical flower-like gold nanostructure/MXene/polypyrrole (HFGNs/MXene/PPY) genosensor for rotavirus RNA detection by employing a particular ssDNA probe and methylene blue as a redox marker. The sensor had a detection limit of about 0.8 a.m., a determination range of 10^−18^–10^−7^ M, high selectivity and stability.

Despite these benefits, there are still challenges for clinical translation, including variability in synthesis and surface chemistry, and oxidation during storage and operation, as well as potential for non-specific adsorption and biofouling in complex clinical samples. Thus, there is a need for improved reproducibility, long-term stability, and standardized surface functionalization and validation with authentic clinical samples (Majeed et al., 2026). The conventional synthesis of MXenes also may use fluoride-containing etchants, posing safety and environmental issues. Electrochemical exfoliation, microwave-assisted etching and molten-salt synthesis are some alternative approaches that could enhance safety and scalability of synthesis (Parihar et al., 2024).

### 4.3 Critical comparison of nanoparticle platforms for viral detection

The limit of detection (LOD) is not the only measure by which to compare nanoparticle platforms for viral detection, as other factors such as particle composition, surface chemistry, recognition elements, assay configuration and sample matrix are all important. For instance, Ru (bpy)_2_^+^ doped silica nanoparticles (SiNPs) were used to detect H9N2 avian influenza with an LOD of 140 fg/mL; on the contrary, a SARS-CoV-2 assay based on UCNPs showed an LOD of 3.59 pg/mL. These values cannot be compared directly due to differences in targets and assay conditions.

ODFNs are characterized by high signal amplification and easy to functionalize; however, dye loading, particle properties and optical detection control is required. MNCs are tunable fluorescent and highly sensitive, but the performance and reproducibility are highly dependent on the composition of the ligand and the synthesis conditions. The synthesis and fabrication of UCNPs are more complex, but they offer low background, photostable detection. Although analytical sensitivity is often lower, AuNPs have well-developed surface chemistry, easy-to-use visual readouts, proven lateral flow formats, and reasonably promising manufacturing capabilities. Magnetic nanoparticles are also of great importance for viral enrichment and separation and could be used with extra instrumental requirements.

Importantly, the specificity is not determined by the core of the nanoparticle but mostly by the recognition element. Stability, reproducibility, toxicity, manufacturing complexity, cost, clinical validation, and regulatory feasibility must therefore be evaluated in conjunction with analytical performance, from a translational point of view. This multi-dimensional assessment is especially important because a large number of viral biosensors based on nanoparticles are still at the proof-of-concept stage and matrix effects, scalability, stability and regulatory issues hinder their use more broadly. However, Table 3 is not a standardized quantitative score, but rather a qualitative, comparative representation of the relative maturity of the translations and technologies. Comparisons of reported LODs should not be made between studies due to differences in viral targets, sample matrices, recognition elements, assay formats and measurement conditions.

**TABLE 3:** Critical comparison of major nanoparticle platforms used in viral diagnostics.

Nanoparticle platform	Principal analytical advantage	Sensitivity potential	Specificity	Stability	Toxicity/safety considerations	Manufacturing/reproducibility	Clinical translation/commercialization potential
AuNPs	Colorimetric signal; simple visual readout; strong surface chemistry	Moderate–high; enhanced by signal amplification and hybrid systems	High when coupled with specific antibodies/aptamers/nucleic-acid probes	Generally good; aggregation and surface chemistry require control	Generally favourable for diagnostic/external use; systemic exposure requires product-specific assessment	High relative maturity; comparatively established synthesis and LFIA integration	High relative potential, particularly for lateral-flow and POC formats
AgNPs/Ag nanoclusters	Strong plasmonic/fluorescent/catalytic properties	High in optimized assays	High with appropriate recognition elements	Sensitive to oxidation, aggregation and ligand environment	Greater concern regarding ion release and cytotoxicity than Au-based systems	Moderate; synthesis and surface chemistry can influence reproducibility	Moderate for diagnostics; therapeutic translation remains more restricted by safety considerations
ODFNs	High fluorophore loading and signal amplification	Very high; femtogram-level detection reported	High with appropriate biorecognition molecules	Improved over free dyes, but dye leakage/loading and photostability require control	Dependent on polymer/silica composition and dye chemistry	Moderate; reproducible encapsulation and dye loading must be controlled	Moderate–high for *ex vivo*/diagnostic applications
Metal nanoclusters (Au/Ag/Cu)	Tunable fluorescence, catalytic and electrochemical signals	Very high in optimized molecular assays	High when coupled with sequence-specific recognition	Strongly dependent on ligand shell and synthesis conditions	Metal-specific toxicity and long-term fate require assessment	Moderate–low compared with mature AuNP systems; precise synthesis required	Moderate; currently stronger as research platforms than established clinical products
UCNPs	NIR excitation; low autofluorescence; photostable quantitative signal	Very high	High with antibody/aptamer/nucleic-acid recognition	High photostability	Composition, surface coating, biodistribution and long-term toxicity require evaluation	Moderate–low; dopant composition and surface modification require tight control	Moderate; attractive for high-performance POC assays but less mature commercially
Magnetic nanoparticles	Target enrichment, separation and concentration; compatible with integrated biosensors	High when coupled with downstream signal amplification	High with appropriate capture ligands	Generally good, but surface coatings are critical	Iron oxide systems have comparatively favourable biomedical profiles; coating-dependent effects remain	Moderate–high; scalable synthesis is possible but device integration adds complexity	Moderate–high for sample preparation and integrated POC systems
Quantum dots	Broad excitation, tunable emission, high brightness and multiplexing	Very high	High with suitable biorecognition	High photostability	Composition-dependent; cadmium-containing QDs raise particular toxicity concerns	Moderate; synthesis and surface passivation require stringent control	Moderate for *ex vivo* diagnostics; more limited for systemic/*in vivo* applications
MXenes/MXene composites	High conductivity, large surface area, suitable for electrochemical and biosensing formats	High; but few reported low detection limits	High when coupled with specific antibody/aptamer/nucleic-acid	Oxidation and surface chemistry changes require control	Safety assessment required	Batch variability, oxidation and synthesis conditions remain concerns	Promising for portable and point-of-care detection, but clinical validation remains limited

### 4.4 Raman spectroscopy and surface-enhanced Raman spectroscopy for viral diagnostics

Raman spectroscopy offers a molecular fingerprint, based on the vibrational properties of the viral components, such as proteins, lipids, nucleic acids, etc., and has been identified as a promising technique for viral diagnostics. The lack of a label confers an advantage for direct biochemical characterization, but the weakness of the Raman scattering makes it difficult to detect at low viral concentrations. This can be circumvented by using surface-enhanced Raman spectroscopy (SERS), which exploits the local electromagnetic field generated by plasmonic nanoparticles, such as Au and Ag nanoparticles, to enhance Raman signals by a factor of many times (Chaudhary et al., 2022; Lukose et al., 2023; Yilmaz et al., 2026).

Label-free fingerprinting, affinity-based assays, Raman nanotags and lateral-flow formats have all found applications for the detection of viruses with the use of nanoparticles. By binding antibodies, aptamers, receptors, or nucleic-acid probes, Au or Ag nanostructures can selectively recognize viruses; additional Raman reporter molecules can enhance the discrimination of signals. Magnetic–plasmonic systems additionally bring component of the target enrichment with the Raman signal amplification. For instance, magnetic SERS nanotags are used as lateral flow assays for H1N1 detection, which reportedly detects 50 PFU/mL of H1N1, and for detection of adenoviruses.

SERS has shown promise in detecting clinically relevant viruses such as SARS-CoV-2, influenza, HIV, hepatitis B and C viruses and HPV. Label-free SERS has been used to discriminate viral infections from complex biological samples and affinity-based substrates have been used to achieve selective detection of viral proteins or particles. Combined with machine learning algorithms, SERS has been used to quickly distinguish influenza from other respiratory viruses, showing the potential of combining the Raman signals of nanoparticles with computational classification (Tabarov et al., 2022; Yang L et al., 2022; Lv et al., 2023). These methods have also been extended to clinical samples and multiplexed detection with recent studies, further demonstrating the potential of SERS for rapid point-of-care and outbreak surveillance applications.

Machine learning (ML) holds special significance for the label-free Raman diagnostics as the viral spectral region contains overlapping biochemical signatures, which can be hard to interpret based on individual Raman peaks. Multidimensional spectral pattern identification and enhancement of discrimination between viruses or viral strains can be obtained using algorithms like support vector machines, principal component analysis, random forests, and other classification algorithms (Tabarov et al., 2022; Yang Y et al., 2022). Such a combination of SERS and ML could then represent a pathway to fast, reagent-less and, potentially, multiplexed viral diagnostics.

All these gains have not yet been translated into routine clinical use. The performance of SERS can be influenced by the properties of the nanoparticles, including their composition, morphology, the distribution of hotspots, the surface chemistry, and measurement conditions. Biological matrices can also cause non-specific adsorption and spectral interference. With label-free approaches, powerful spectral preprocessing is necessary, acquisition protocols must be standard, and training sets of sufficiently high diversity must be used to achieve reproducibility between instruments, laboratories, and patient groups (Lukose et al., 2023; Chaudhary et al., 2022). Reproducible plasmonic substrates, portable Raman instrumentation, microfluidics and lateral-flow integration, magnetic target enrichment, multiplexed detection, and AI-assisted spectral analysis are important areas for future development. New techniques like near-infrared Raman spectroscopy (NIR-SERS), tip-enhanced Raman spectroscopy and coherent Raman techniques could further enhance the analytical power of Raman based virology (Yilmaz et al., 2026).

## 5 Nanoparticles as antivirals

Silver NPs are a well-researched class of antiviral NPs, and unmodified and surface-modified AgNPs have been shown to neutralize a wide range of viral pathogens (Rai et al., 2014). This mode of therapy is especially effective against highly mutagenic viruses, as it prevents the development of viral resistance. AgNPs not only have an extracellular effect by blocking virion entry but also an intracellular effect by inhibiting viral replication. In a cellular assay, commercially prepared AgNPs with a diameter of 30–50 nm were shown to bind the gp120 envelope glycoprotein of HIV-1, disrupt gp120-CD4 interactions in a dose-dependent manner, and prevent viral fusion (Lara et al., 2010). Furthermore, AgNPs have also been used in combination with broadly neutralizing antibodies to cure HIV (Lara et al., 2011; Singh and Lara, 2012). It was found that nascent 10 nm AgNPs were effective in preventing hemagglutination caused by the H1N1 influenza A virus *in vitro* (Xiang et al., 2011). Also, AgNPs were observed to inhibit intracellular Hepatitis B virus (HBV) replication, presumably through direct contact with viral DNA (Lu et al., 2008; Sun et al., 2005). Representative metal-based NPs exhibiting antiviral activity, along with their mechanism and outcomes, are summarized in Table 4.

**TABLE 4:** Metal-based nanoparticles with antiviral properties.

Nanoparticle type	Size range	Target Virus/System	Mechanism of antiviral action	Outcome	Citation
Gold nanoparticles (AuNPs)	10–60 nm	Barley viruses	Capsid disruption	*In vitro* disassociation of viral particles	(Aref et al., 2012; Alkubaisi and Aref, 2017)
Zinc oxide nanoparticles (ZnO NPs)	15–70 nm	Tobacco and cucumber viruses	Capsid disruption through the production of reactive oxygen species (ROS)	Deactivation of TMV particles and a corresponding decrease in the severity of the infection	(Cai et al., 2019; El-Sawy et al., 2017)
Silica nanoparticles (SiO_2_ NPs)	∼10–50 nm	Tomato and cucumber viruses	Resistance pathways are activated, structural interference which inhibits viral replication	Decline in viral load and reduction in the symptoms of the disease	(El-Shazly et al., 2017; Elsharkawy and Mousa, 2015)
Iron oxide nanoparticles (Fe_2_O_3_/Fe_3_O_4_)	10–50 nm	Tobacco and turnip viruses	The activation of salicylic acids defense and the upregulation of antioxidative enzymes	Inhibition of viral growth and its geographic distribution	(Cai et al., 2020)
Titanium dioxide nanoparticles (TiO_2_ NPs)	70–100 nm	Bean virus	ROS-mediated antiviral action	Reduction in the severity of the diseases in Vicia faba	(Elsharkawy and Derbalah, 2019)

Rigidly varying numbers of other antiviral NPs of metals and their compounds have been documented (Alghrair et al., 2019; Rai et al., 2016). HBV infection has been suppressed using AuNPs loaded onto layered double hydroxide substrates (Carja et al., 2015). Copper iodide NPs produce reactive oxygen species and oxidize capsid proteins, preventing the swine H1N1 influenza virus (Fujimori et al., 2012) and feline calicivirus (Shionoiri et al., 2012). Recently, copper oxide NPs were used to treat HSV-1 infections and reduce the viral load by up to 83.3% (Tavakoli and Hashemzadeh, 2020). Titanium dioxide NPs show similar effectiveness against the H3N2 influenza virus (Mazurkova et al., 2010), but iron oxide NPs are effective against the H1N1 influenza virus (Kumar et al., 2019). The heavy metal gallium demonstrated the ability to prevent HIV and *Mycobacterium tuberculosis* infections as NPs (Narayanasamy et al., 2015). Silica nanoparticles (SiNPs) prevent HIV and respiratory syncytial virus (RSV) infection at 0.1 mg/mL and 0.01 mg/mL concentrations, respectively (Osminkina et al., 2014).

AgNPs can also prevent a variety of viral infections by inhibiting viral entry into cells or killing extracellular virions, such as the herpes simplex virus (HSV), RSV, and adenovirus type 3 (Baram-Pinto et al., 2009; Morris et al., 2019). The widespread antiviral activity of AgNPs results from their large surface area, which enables them to interact with the viral particles. Antiviral agents may act directly on the virus, such as by attaching to the viral protein coat and preventing the virus from binding or performing other functions. First, AgNPs interact with the viral surface, then affect the viral genomic material, or inhibit its ability to puncture the host cell membrane. AgNPs alter the interactions between virions and cell membranes, inhibit nucleocapsid disassembly, and disrupt the integrity of the viral genome, thereby inhibiting viral replication in host cells. These nanoparticles disrupt key biological functions, including protein synthesis, thereby hindering viral replication (Table 5). Although other metallic nanoparticles also possess antiviral properties, AgNPs are the most potent, demonstrating strong efficacy against viral families (Ratan et al., 2021).

**TABLE 5:** Antiviral actions of AgNPs.

Virus	AgNP type	Antiviral mechanism	Citation
HIV-1	Uncoated and PVP-coated AgNPs	The gp120-CD4 binding is blocked, preventing viral entry	(Elechiguerra et al., 2005; Lara et al., 2010)
HBV	HEPES-buffer synthesized AgNPs (10–50 nm)	HBV DNA binding leads to the inhibition of the synthesis of pgRNA and genomic replication	(Lu et al., 2008)
HSV-1	MES-coated and uncoated AgNPs	Viral adherence to host cells can be prevented by mimicking heparan sulphate	(Baram-Pinto et al., 2009)
Influenza A Virus	Uncoated AgNPs (5–20 nm)	The secondary effect of inhibition of hemagglutination is that it suppresses apoptosis	(Xiang et al., 2011)
RSV	Curcumin-coated AgNPs	The concomitant inhibition of the viral entry is observed on direct inactivation of virions	(Yang Y et al., 2022)
Poliovirus	Electrochemical AgNPs (4–9 nm)	Prevention of attachment of virions to cellular receptors suppresses further infection	(Speshock et al., 2010)
Chikungunya Virus	Plant-based green AgNPs	Viral replication is suppressed, hence lowering the viral load	(Sharma et al., 2021)
SARS-CoV-2	Photochemical AgNPs (10–30 nm)	Viral entry is inhibited directly by inactivating virions	(Stanley, 2020)
WSSV	PVP-coated AgNPs (∼35 nm)	Viral replication can be reduced by activation of the immune receptors	(Ochoa-Meza et al., 2019)

The antiviral activity of the nanoparticles is related to their effectiveness, compatibility with the host cells, stability, and processability. AgNPs are found to be broadly antiviral, however, their size, coating, dosage, and exposure time must be well controlled, as they may be toxic to the host cell. The AuNPs can also be functionalized for a wide range of applications and may be used as antiviral agents or targeted delivery systems, but biodistribution and clearance must be taken into account. The reactive oxygen species and surface interactions of ZnO, CuO, and TiO_2_ nanoparticles have an antiviral effect and may have a more limited therapeutic window. Amongst nanoparticles, silica-based and polymeric nanoparticles are of special interest for controlled drug delivery due to their loading and release capabilities, while scaling up and reproducibility are challenges. No single platform is superior, so the choice should be based on the application, safety, manufacturability, therapeutic index, and clinical benefit.

## 6 Virus-templated synthesis of nanoparticle

Within the nanotechnology domain, science, especially health sciences, electronics, and environmental studies, has changed drastically. AgNPs are among the most commonly used nanomaterials because of their unique antibacterial, optical, and catalytic properties. Traditional synthetic methods for AgNPs generally utilize a palette of physical and chemical methods; nevertheless, these methods often utilize toxic agents and require a lot of energy (Dhaka et al., 2023). Another environmentally friendly alternative is biogenic synthesis, in which living organisms, such as plants, fungi, bacteria, and viruses, serve as nanofactories (Ahmad et al., 2024). Among others, viruses have attracted the attention of scholars because of their well-defined nanostructures, intrinsic self-assembly properties, and innate biocompatibility (Ahmad et al., 2024; Dhaka et al., 2023).

A virus-mediated process occurs through biomineralization, in which viral capsids play a major role in nucleating and growing NPs (Dhaka et al., 2023). Silver cation (Ag^+^) binding to capsid proteins is also a major event in this process and in the antiviral effect of silver. The capsid also possesses several ligand-binding sites, such as primary amines (–NH2), thiols (–SH), hydroxyls (-OH), and carboxylates (-COO-), all of which promote Ag^+^ binding (Hangyu et al., 2024). This is followed by the reduction of Ag^+^ to elemental silver (Ag^0^) mediated by either capsid proteins or the associated nucleic acid, thus triggering the *in situ* generation of NPs. This mechanistic pathway is biogenic, in which macromolecular constituents serve as reductants. Environmental factors that influence the effectiveness of the reduction step include pH, temperature, and light exposure (Mikhailova, 2020).

### 6.1 Mechanism of virus-mediated nanoparticle synthesis

The mechanism of virus-mediated nanoparticle synthesis, as illustrated in Figure 3, involves the specific interaction of viral surface proteins or capsid structures with metal ions, facilitating their nucleation, reduction, and controlled assembly into stable nanoparticles.

**FIGURE 3:**
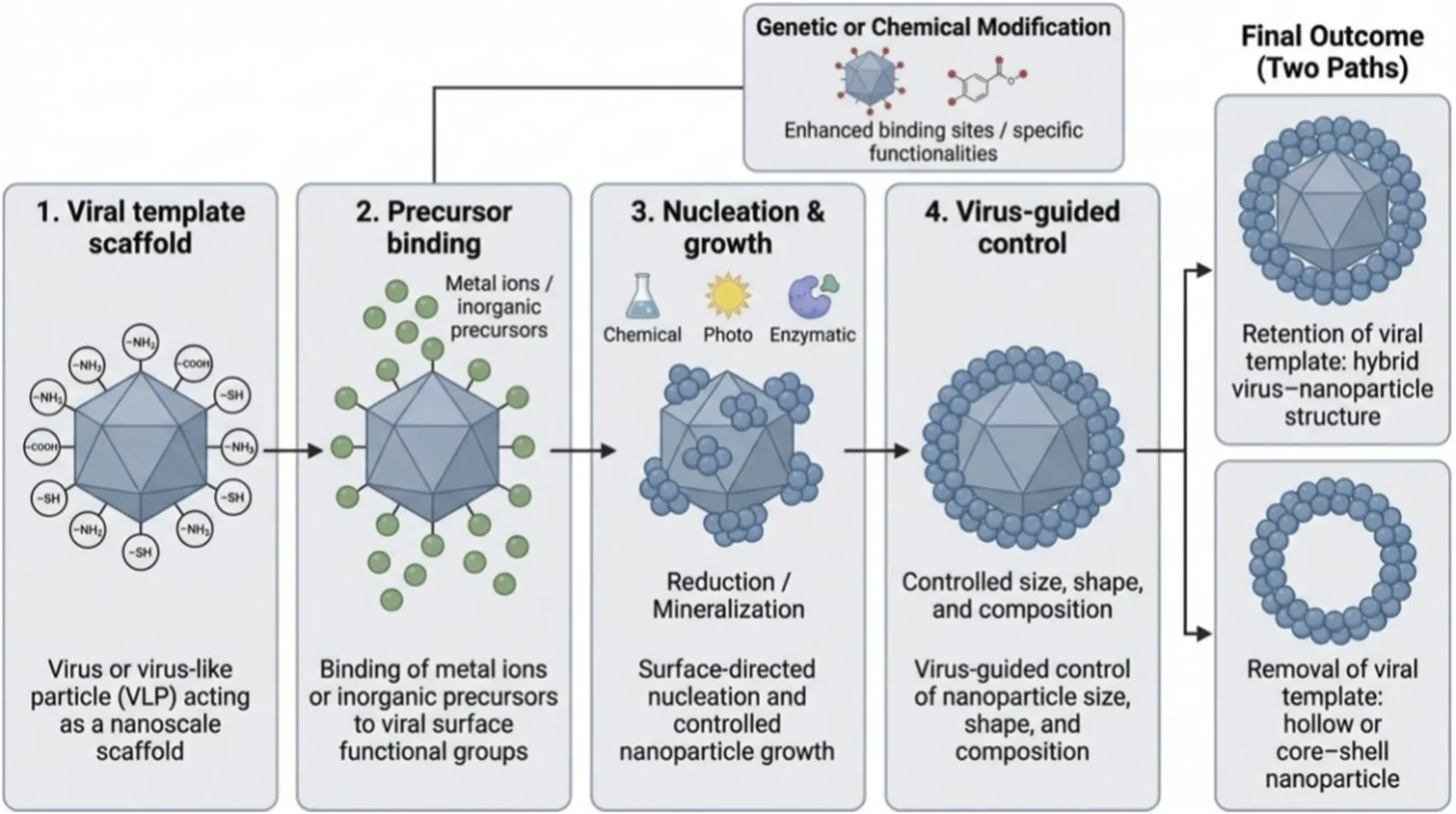
Mechanism of virus-mediated nanoparticle synthesis.

#### 6.1.1 Virus as scaffold/template

Viruses (or virus-like particles, VLPs) provide a highly ordered, monodisperse, and modifiable scaffolding. Their protein shells present functional groups (e.g., carboxyl, amine, and thiol) on the inner and/or outer surfaces, which serve as nucleation sites for inorganic material deposition. (Cumbo et al., 2013; Lee et al., 2021; Li and Wang, 2014; Liu et al., 2017; Mao et al., 2004; Telkapalliwar and Deharkar, 2025; Zhou et al., 2014).

#### 6.1.2 Nucleation and growth

Metal ions or precursors are introduced and bind to these functional groups via electrostatic interactions or covalent bonds. Reduction (chemical, photochemical, or enzymatic) or mineralization then initiates nucleation directly on the virus surface, rather than in bulk solution, leading to the controlled growth of NPs (Atanasova et al., 2017; Liu et al., 2017; Moreira Da Silva et al., 2023; Slocik et al., 2005; Telkapalliwar and Deharkar, 2025).

#### 6.1.3 Control of morphology and composition

The spatial arrangement and density of nucleation sites on the virus determine the size, shape, and NP distribution. The genetic or chemical modification of the virus can further tune these properties, enabling the synthesis of core-shell, hybrid, or composite nanostructures (Atanasova et al., 2017; Cumbo et al., 2013; Lee et al., 2021; Li and Wang, 2014; Liu et al., 2017; Mao et al., 2004; Zhou et al., 2014).

#### 6.1.4 Removal or retention of template

The viral scaffold can be retained for hybrid materials or removed (e.g., by thermal degradation) to yield hollow or core-shell NPs (Liu et al., 2017; Zhou et al., 2014).

After reduction, coaggregation and co-nucleation of silver atoms on the viral surface result in the formation of NPs with a highly defined morphology. An example of the deposition of AgNPs on a viral scaffold can be viewed as an iconic biotemplated nanomaterial (Ivanov et al., 2023).

Plant viruses have a highly symmetrical protein capsid, making them excellent bio-templates for AgNP biosynthesis (Jain et al., 2021). Their naturally stable nanostructures, exquisitely defined surface chemistries, and inherent stability provide a superior scaffold for regulating the nucleation and growth of AgNPs. Icosahedral or rod-shaped capsids with a self-assembly type are usually applied to obtain NPs with identical sizes and shapes. Moreover, they are nature-based and biocompatible viruses that facilitate green chemistry in the production of AgNPs and eliminate the use of harsh chemical reductants (Jain et al., 2021). Viruses of plants utilized in the production of AgNPs include the tobacco mosaic virus (TMV), cowpea mosaic virus (CPMV), brome mosaic virus (BMV), and potato virus X (PVX).

Plant viruses are organized production systems that template AgNPs. Their unique capsid cubicity, functional group distribution, and biocompatibility help regulate the composition of NP assemblages, enhancing their potential applications in medicine, electronics, and materials science (Telkapalliwar and Deharkar, 2025).

Some animal viruses, such as bacteriophage M13 and Newcastle disease virus (NDV), have also been explored for the synthesis of AgNPs because of their tendency to self-assemble, applicability as biological templates, and well-defined capsid structures (Kakakhel et al., 2021; Scibilia et al., 2016).

Nanoparticles generated using this approach offer unique benefits compared to those generated using other chemical and physical techniques, making it an appealing strategy for producing nanomaterials. The ultimate advantage of biotemplating is its targetability: viruses can control the construction of highly ordered monolithic nanostructures, enabling nucleation and growth, and producing monodisperse NPs with a predefined shape and size (Jeevanandam et al., 2022). Furthermore, it is a benign, environmentally friendly, and non-toxic method of synthesizing NPs that rejects the use of debilitating reducing agents and harsh reaction conditions, thereby advancing the paradigm of green nanotechnology (Telkapalliwar and Deharkar, 2025).

AgNPs synthesized through virus templating are more stable and dispersible because aggregation is prevented by the viral scaffold, thus extending the functional stability of the NPs. In addition, the self-assembly of viruses enables the fabrication of complex structures, such as nanowires and nanocages, thereby extending their use in areas such as nanoelectronics, catalysis, and environmental cleanup (Telkapalliwar and Deharkar, 2025).

## 7 Advancement in nanoparticle-mediated viral inhibition

Nanoparticles have emerged as promising antiviral agents because of their unique physicochemical properties, which allow them to interact with viruses in ways that traditional drugs cannot. AgNPs have demonstrated broad-spectrum antiviral activity against a range of viruses, including those affecting humans and animals, through mechanisms such as direct virion destruction and inhibition of viral entry; however, challenges remain in ensuring safety and minimizing toxicity to host cells (Haggag et al., 2019; Janicka et al., 2025; Ratan et al., 2021).

Zinc oxide NPs (ZnO-NPs) have also shown potent antiviral effects, particularly against influenza and herpes simplex viruses, with PEGylated forms achieving high inhibition rates and reduced viral loads *in vitro* and in animal models (Attia et al., 2021; Ghaffari et al., 2019; Melk et al., 2021).

AuNPs are notable for their versatility, being used not only for direct antiviral effects but also as carriers for antiviral drugs and vaccine antigens, and have demonstrated efficacy against viruses such as SARS-CoV-2, HSV-1, and influenza in both *in vitro* and *in vivo* studies (Babaei et al., 2021; Elste et al., 2023; Ren et al., 2023; Teimouri et al., 2025).

NPs can enhance the delivery and efficacy of existing antiviral drugs by improving their solubility, bioavailability, and targeted release, thereby reducing side effects and overcoming issues such as drug resistance (Delshadi et al., 2021; Lembo et al., 2018; Wang et al., 2023). For example, NP-encapsulated antiviral agents, such as favipiravir and oseltamivir, have shown improved therapeutic outcomes in animal models of neurotropic and respiratory viral infections, including increased survival rates and reduced viral titers (Hu et al., 2018; Ren et al., 2023; Wang et al., 2023). Additionally, functionalized NPs, such as those modified with tannic acid or loaded with natural prodrugs, can modulate immune responses and provide synergistic antiviral effects (Abouaitah et al., 2020; Díaz et al., 2025; Janicka et al., 2025).

### 7.1 MXenes as antiviral nanomaterials

The surface area, electrical conductivity, hydrophilicity and chemically active surface of MXenes make them attractive for biomedical applications. They are typically illustrated as having the general formula M_n+1_X_n_T_x_, where M is a transition metal, X is a carbon and/or a nitrogen, and T_x_ is a surface termination group such as (–O, –OH, and–F). These surface groups facilitate interactions with biomolecules and enable a “tuned” surface property of MXenes depending on its intended application (Huang et al., 2021). Additionally, the surface chemistry, conductivity, and two-dimensional structure of MXenes have led to their application in antiviral sensing and other biomedical uses (Sarac et al., 2025).

The direct antiviral activity of MXenes has not been studied as extensively as that of metallic nanoparticles and lipid-based systems. Unal et al. (2021) compared the susceptibility of Ti_3_C_2_T_x_, Ta_4_C_3_T_x_, Mo_2_Ti_2_C_3_T_x_ and Nb_4_C_3_T_x_ to various SARS-CoV-2 genotypes. The reduction in viral infection was significantly greater in Vero E6 cells infected with the GR clade in the presence of Ti_3_C_2_T_x_, but not the same in the viral genotypes tested. The antiviral activity was exhibited by Mo_2_Ti_2_C_3_T_x_, while Ta_4_C_3_T_x_ and Nb_4_C_3_T_x_ did not demonstrate a significant antiviral effect under the test conditions (Unal et al., 2021). In the same study, Ti_3_C_2_T_x_ gave greater than 99% inhibition at the reported dilution, with an IC_50_ of 0.32 μg/mL while Mo_2_Ti_2_C_3_T_x_ gave more than 95% inhibition at 100 μg/mL. These results also revealed that the antiviral activity was genotype- and composition-dependent of the viruses and MXenes, respectively, suggesting that the activity of the MXenes should not be taken as being the same for all viral variants and the same for all viruses.

The mechanism behind viral inhibition by MXene is not yet understood, but it is likely that the adsorption of viral particles, interaction with viral surface proteins, or structural changes in proteins that possibly disrupt virus-host receptor interactions are responsible for the inhibition. Ghasemy et al. (2022) used an *in silico* approach to investigate the interaction between the SARS-CoV-2 spike protein and the MXenes Mn_2_C, Ti_2_C and Mo_2_C. The modelled MXenes were predicted to interact with the spike protein and showed potential to alter its secondary-structure characteristics, potentially disrupting subsequent spike protein–ACE2 binding. The observations were derived from computational modelling and should therefore be regarded as mechanistic hypotheses rather than direct proofs of antiviral activity in biological systems.

The surface chemistry of MXenes is also a basis of their antiviral activity. Hydrophilicity, adsorption, interactions with biomolecules and the possibility of further surface modification is affected by the–O, –OH and–F surface groups. Surface engineering can thus be utilised to manipulate dispersion, stability, and biological interactions. The aggregation of unmodified MXenes under physiological conditions, however, has been studied and the possibility to surface modify them to promote their stability and biological functionality has been explored (Huang et al., 2021). Functionalization, doping, and synthesis of composites comprising 2D nanomaterials have also been highlighted in recent reviews as potential means of modulating their antiviral effects, but much remains at the laboratory and proof-of-concept levels (Chowdhury et al., 2025).

While promising, there are a number of challenges to be addressed before MXenes could be used in a wider range of virological applications. The biological performance may differ when they vary in composition, flake size, surface termination, aggregation state, and surface modification. Concentration and material properties also may play a role in toxicity. Furthermore, existing research has predominantly focused on short-term effects, and the degradation and fate of MXenes and their products in biological systems remain poorly understood (Huang et al., 2021; Chowdhury et al., 2025). Another factor that affects the stability and reproducibility of MXene-based systems is oxidation and changes in surface chemistry. The other factor that affects the stability and reproducibility of MXene-based systems is oxidation and changes in surface chemistry. Thus, well-characterized MXenes, including suitable toxicity controls, should be used in future studies, as should at least different viral genotypes, and antiviral effects should be validated in physiologically relevant models. The evidence is currently positive for using MXenes as platforms for research on antiviral materials, and more experimental and translational research is needed for their antiviral use to be considered mature.

### 7.2 Lipid nanoparticles beyond vaccine delivery

Beyond their role in mRNA vaccines, lipid nanoparticles (LNPs) have proven to be versatile platforms in virology, with the ability to encapsulate nucleic acids, shield them from degradation, and promote their cellular uptake and subsequent release inside cells. LNPs are illustrated in the clinical translation of LNP-formulated siRNA therapeutic patisiran, which shows the potential of systemic RNA interference (Adams et al., 2018; Kulkarni et al., 2021; Cullis and Felgner, 2024). For antiviral applications, LNPs can be used to deliver siRNA sequences targeting conserved virus transcripts or host factors that play a role in viral replication; however, sequence specificity and genetic variation of the virus should be considered (Idres et al., 2025).

LNPs are also being explored as non-viral vectors for CRISPR therapeutics. Cas9 mRNA and guide RNA delivery can result in transient genome-editing activity, while selective-organ-targeting (SORT) approaches have shown that lipid composition can affect tissue distribution, which allows for tissue-specific editing (Qiu et al., 2021; Cheng L et al., 2020). In antiviral applications, these systems could eventually be directed at viral dependency factors, reservoirs, or viral genome, but duration of editing, immunogenicity, off-target effects and tissue specificity should be considered.

In addition to nucleic acids, lipid-based nanocarriers can also enhance the solubility, stability, pharmacokinetics, and targeted delivery of conventional antiviral drugs (Maithania et al., 2026). New and novel approaches to lipid formulation and targeting will also pave the way for individual tailored antiviral nanomedicine based on viral genotype, infected tissue and host characteristics, albeit personalized LNP-based antiviral therapy is still an embryonic clinical strategy (Dilliard and Siegwart, 2023; Vasileva et al., 2024). Table 6 shows the NP-based antiviral nanomedicines and nanovaccines that are available or under evaluation, highlighting their modes of action, target viral infections, and advantages. The content in Table 6 are grouped by their degree of maturity, to differentiate the translations on the various platforms mentioned. Products that are approved/licensed, regulatory approved vaccines, clinical stage products, withdrawn clinical stage products and preclinical/experimental vaccines are thus distinguished.

**TABLE 6:** Nanoparticle-based antiviral nanomedicines and nanovaccines that are available or under evaluation.

Category	Nanoplatform type	Composition/Material	Mechanism of action	Target viral infection	Specific medicine/Vaccine	Development stage*	Key advantages	Citation
Nanomedicine	Liposomes	Phospholipid bilayer vesicles	Encapsulation and controlled antiviral drug release; membrane fusion	HIV, Influenza, HSV	Liposomal acyclovir, Liposomal Zidovudine	Preclinical/experimental formulation	Reduced toxicity and enhanced delivery	Lembo et al. (2018)
​	Polymeric Nanoparticles (PLGA/PLA)	Biodegradable polymers	Sustained intracellular drug delivery	HIV, Hepatitis B/C	Nano-Tenofovir, Nano-Ritonavir	Preclinical/experimental formulation	Prolonged circulation and targeting	Cojocaru et al. (2020)
​	Solid Lipid Nanoparticles	Lipid-based nanocarriers	Improved permeability and drug stability	Respiratory viral infections	SLN-based Efavirenz	Preclinical/experimental formulation	Enhanced bioavailability	Lembo et al*.* (2018)
​	Dendrimers	Branched polymer nanoparticles	Viral attachment and entry inhibition	HIV, HSV	VivaGel® (SPL7013)	Marketed/registered for selected non-antiviral indications; antiviral application not established as an approved therapeutic	Intrinsic antiviral activity	Singh and Lara (2012)
​	Metallic Nanoparticles	Silver/Gold nanoparticles	Interaction with viral surface proteins	Influenza, Dengue, HIV	Silver nanoparticle antiviral systems	Preclinical/experimental	Direct virucidal effect	Singh and Lara (2012)
​	Carbon-based Nanomaterials	Graphene oxide, CNTs	Viral membrane disruption and drug transport	SARS-CoV-2, Influenza	Functionalized graphene carriers	Preclinical/experimental	Large surface area targeting	Tonelli et al. (2015); Dash and Kundu (2023)
​	Nanoemulsions/Micelles	Surfactant nano-systems	Solubility enhancement of antivirals	HSV, Hepatitis	Micellar acyclovir	Preclinical/experimental formulation	Increased permeability	Lembo et al. (2018)
​	siRNA Nanocarriers	Lipid/polymer hybrid nanoparticles	Gene silencing of viral replication	Ebola, HIV, SARS-CoV-2	TKM-Ebola (siRNA nanoformulation)	Clinical trial—Phase II; development discontinued	Target-specific antiviral therapy	Chakravarty and Vora (2021)
​	Lipid nanoparticles	Ionizable lipids, helper phospholipids, cholesterol, PEG-lipid	Transient delivery of Cas9 mRNA and sgRNA for CRISPR-mediated genome editing	Experimental applications against persistent viral genomes and host dependency factors	Cas9 mRNA/sgRNA LNP systems	Preclinical/experimental	Non-viral delivery, transient expression, and potential tissue-selective targeting	Cheng L. et al. (2020); Qiu et al. (2021)
​	Lipid-based nanocarriers	Phospholipids, cholesterol and lipid-based carrier components	Encapsulation, controlled release, and enhanced cellular/tissue delivery of antiviral drugs	Respiratory and other viral infections	Lipid-based antiviral drug formulations	Experimental/emerging platform	Improved drug stability, bioavailability, pharmacokinetics and targeted tissue exposure Lembo et al. (2018)	Maithania et al. (2026)
​	Organ-/cell-targeted LNPs	Ionizable lipids with tunable lipid composition and targeting components	Modulation of tissue distribution and selective delivery of therapeutic cargo	Emerging applications across virus-specific and host-directed antiviral therapies	Programmable LNP-based RNA/drug delivery systems	Preclinical/experimental	Potential for patient-, tissue-, and payload-specific antiviral treatment	Dilliard and Siegwart (2023); Vasileva et al. (2024)
Nanovaccine	Lipid Nanoparticle (LNP) Vaccines	Ionizable lipid nanoparticles	mRNA protection and antigen delivery	COVID-19	BNT162b2 (Pfizer-BioNTech), mRNA-1273 (Moderna)	Approved/licensed as Comirnaty and Spikevax	Strong humoral and cellular immunity	Heng et al. (2022)
​	Protein Nanoparticle Vaccine	Recombinant spike protein nanoparticles	Enhanced antigen presentation	SARS-CoV-2	NVX-CoV2373 (Novavax)	Approved/licensed as Nuvaxovid	High immunogenicity	Montastruc et al. (2022)
​	Virus-Like Particle (VLP) Vaccine	Self-assembled viral proteins	Mimics virus without genome	HPV, Hepatitis B	Gardasil, Engerix-B	Approved/licensed	Excellent safety and immune response	Cheng Q. et al. (2020); Mironova and Ghany (2024)
​	DNA Nanovaccine	Plasmid DNA nanoparticles	Host antigen expression	COVID-19	ZyCoV-D	Regulatory authorisation (India; EUA)	Stable and needle-free delivery	Silveira et al. (2021); Heng et al. (2022)
​	Peptide-based Nanovaccine	Epitope-loaded nanoparticles	B-cell and T-cell activation	Zika, HIV	Multi-epitope peptide nanovaccines	Preclinical/experimental	Specific immune targeting	Heng et al. (2022)

* Development stage refers to the regulatory or translational status of the representative product/formulation rather than the maturity of the underlying nanoparticle platform. “Approved/licensed” denotes authorization for clinical use for the stated indication; “Regulatory authorization (EUA)” denotes emergency-use authorization; “Clinical trial—discontinued” denotes a candidate that entered human clinical evaluation but whose development was subsequently discontinued; and “Preclinical/experimental” denotes formulations or platforms without established regulatory approval for the indicated antiviral application. Regulatory status may differ across jurisdictions.

In the context of plants, while the reviewed papers focus primarily on human and animal applications, the principles of NP-mediated viral inhibition, such as gene silencing and targeted delivery, are being explored for plant viral protection, although commercial products are still in development. Despite robust preclinical evidence supporting the antiviral potential of NPs, most NP-based antiviral therapeutics remain in the research or early clinical trial stages, with only a few diagnostic or adjunctive products available in the market. Key challenges for broader market availability include ensuring safety and scalability, obtaining regulatory approval, and demonstrating clear advantages over existing therapies (Delshadi et al., 2021; Lembo et al., 2018; Teimouri et al., 2025).

A separate consideration should be given to the distinction between technical feasibility, clinical translation and regulatory approval. A platform of nanoparticles could have very good analytical performance or antiviral activity, but may have no viable path to product. Regulatory evaluation is typically a product-by-product process, and does not rely exclusively on the identity of the chemical. For instance, in the U.S. Food and Drug Administration (FDA), products with nanomaterials are assessed under a regulatory framework that is appropriate to their intended product category, accounting for their physicochemical properties, safety, effectiveness, and manufacturing attributes (FDA, 2018). The term “clinically approved” should not be used for a specific nanoparticle platform unless the particular product and indication have been shown to be clinically approved. Reproducible control of critical quality attributes such as particle size and distribution, surface characteristics, composition, aggregation state, purity and drug loading is especially important for nanomedicines as small variations in these attributes can impact the biological behaviour.

Commercialization will be more likely to be driven by platforms that provide sufficient analytical sensitivity and low-cost reagents, that are stable at room temperature, and that are easy to use and will be compatible with existing point-of-care formats for viral diagnostics. This is a significant benefit for lateral-flow AuNP-based detection technologies compared to more complex UCNP, MNC and hybrid platforms, which can be valuable for their higher analytical performance, but which might only be clinically relevant if they translate into clinical sensitivity or in multiplexed detection or improved early diagnosis. Translation for therapeutics is much more stringent, as systemic biodistribution, toxicity, pharmacokinetics and manufacturing consistency are also required along with antiviral activity. Therefore, the commercial potential should be assessed by following the entire product-development process and not by the individual performance of the nanoparticles (He Z. et al., 2025).

Overall, the advancement of NPs for viral inhibition is marked by rapid innovation and promising laboratory and animal results; however, translation to widespread clinical or agricultural use is still limited, with ongoing research focused on optimizing efficacy, safety, and delivery systems.

### 7.3 Challenges and translational considerations of antiviral nanomaterials

Although nanomaterial-based antivirals are promising in vitro and in animal models, there are still some obstacles for clinical translation such as toxicity, biodistribution, immune response, biodegradation, long-term safety, manufacture, and regulatory requirements. The toxicity of nanotechnology is determined by morphology, size, aggregation, surface charge, composition, route and duration of administration of the nanotechnology. Given that metallic nanoparticles can cause oxidative stress or release of metal ions; and cationic or ionizable systems can disrupt cellular membranes or signaling, it is recommended that antiviral activity be assessed to account for cytotoxicity, inflammatory responses, genotoxicity, and organ-specific toxicity (Ratan et al., 2021; Shabatina et al., 2022; Bitounis et al., 2024).

Biodistribution and pharmacokinetics are also key because nanoparticles can be retained in organs like the liver or spleen, which can restrict the delivery to the respiratory system, brain or the persistent viral reservoirs. Tissue distribution and cell uptake can significantly vary with lipid composition, particle size, surface properties and administration route (Dilliard and Siegwart, 2023; Vasileva et al., 2024). Nanoparticle-induced immune activation (including complement and inflammatory responses) should also be carefully evaluated, especially for repeated dosing/administration of LNP-based systems (Bitounis et al., 2024). Likewise, biodegradation is not synonymous with safety; degradation products, residual materials or those that have not been cleared effectively may be toxic or are retained in the tissue.

Long term studies are required to determine accumulation, delayed inflammation, immune sensitization, and organ toxicity. There are also formulation dependent differences in the particle size, composition, surface properties, encapsulation efficiency and impurities that further complicate regulatory translation. Standardized characterization, reproducible manufacturing, validated analytical procedures and pharmacovigilance are thus critical. The U.S. FDA has included characterization of the unique physicochemical and biological properties of nanomaterial containing products in its regulatory guidance (FDA, 2018). In summary, future antiviral nanotechnology should encompass the fields of nanotoxicology, pharmacology, virology and regulatory science to create safe, reproducible and clinically translatable platforms.

## 8 Emerging directions in nano-enabled viral diagnostics and therapeutics

The future of nano-enabled virology is anticipated to extend beyond enhancing the sensitivity of nanoparticles to the development of integrated platforms that integrate nanomaterials with artificial intelligence (AI), multiplexed sensing, microfluidics, wearable technologies, digital health, organ-on-chip models, and sustainable synthesis. This convergence may enable rapid detection, decentralized monitoring, and better-informed therapeutic decision making, with the added precondition of appropriate analytical performance, safety, reproducibility and clinical validity.

- AI-assisted diagnostics may enhance interpretation of complex optical, electrochemical, fluorescence, and imaging signals generated by nanobiosensors. While machine-learning algorithms can contribute to the classification of signals, pattern recognition, quantitative prediction, and real-time decision-making, it is crucial to keep in mind several factors of model interpretability, data quality, bias, and generalizability (Akkaş et al., 2025).
- Multiplex and multimodal biosensors could be capable of detecting multiple pathogens simultaneously, including both viruses and antigens, and also nucleic acid targets, and also host biomarkers, with optical, magnetic and plasmonic and electrochemical signatures as complementary detection modalities using nanoparticles. Future systems should be specific, have less cross-reactivity, and perform in a consistent manner in different clinical matrices (He L. et al., 2025).
- The application of MXenes in sensing and antiviral platforms is a new frontier in nano-enabled virology. These properties can enable electrochemical, optical, and other biosensing applications of MXene, and MXenes’ interactions with viral particles and surface proteins can serve as a foundation for exploring antiviral applications. Control of oxidation and surface chemistry, batch-to-batch reproducibility, standardised antiviral testing protocols and evaluation in complex clinical matrices and physiologically relevant models should be the focus of future studies. These studies are important for translating promising laboratory results into reliable diagnostic and antiviral technologies (Huang et al., 2021; Unal et al., 2021; Sarac et al., 2025).
- Microfluidic point-of-care (POC) platforms provide opportunities to incorporate sample preparation, target enrichment, detection and signal generation into sample-to-answer devices to decrease reliance on central laboratories. The integration of upconversion technologies with nanoparticles can further enable fast decentralized viral testing (Kardjadj, 2025; Sekhwama et al., 2024). The wearable sensing platforms can supplement the existing diagnostics and offer longitudinal monitoring of physiological or biochemical changes to transmit data via connected devices. However, given the fact that many of the physiological responses are non-pathogen specific, many wearable systems are likely to remain as monitoring or early-warning systems that will need confirmatory pathogen-specific testing (Ghazizadeh et al., 2024).
- The ability to integrate nano-enabled sensors with smartphones, cloud platforms, and digital health systems may enable real-time data sharing, real-time monitoring, and control of infectious diseases. AI-driven analysis of such data can also play a role in outbreak detection, but there are challenges that need to be tackled, including interoperability, privacy, cybersecurity, and algorithmic transparency (Borham et al., 2025).
- The organ-on-chip platform also has potential for testing antiviral nanomaterials in more physiologically relevant settings by including aspects of tissue architecture, flow, multicellular interactions, and specific immune responses. It is hoped their incorporation with nanobiosensors will allow for real-time monitoring of viral replication, therapeutic responses, distribution of nanoparticles, and toxicity (Alonso-Roman et al., 2024; Zhang et al., 2024).
- Biological or virus-templated synthesis of nanoparticles should be considered alongside aspects of the life cycle of the nanoparticles, such as resources used, scalability, toxicity, waste, and environmental fate. Though large scale manufacturing and reproducibility are challenges, green synthesis exploiting plants, microorganisms, biomolecules and viral templates may alleviate dependence on conventional chemical processes (Jain et al., 2024; Hicks, 2025).

Collectively, these developments suggest that the next-generation of nano-enabled virology will be based on the integration of sensing, computation, microfluidics, digital connectivity, advanced disease models, and sustainable nanomanufacturing, rather than on individual technological improvements.

## 9 Conclusion and future directions

The synergy between the fields of virology and nanotechnology has led to the development of diverse strategies in the field of rapid viral diagnosis, anti-viral therapies and biomimetic synthesis of nanomaterials. Nanomaterials can be used to increase analytical sensitivity, point-of-care testing, targeted delivery, and interfere with multiple steps of the viral life cycle. They are not necessarily ready for use clinically, however, they can perform well on different platforms, and high analytical and antiviral activity do not necessarily mean high clinical activity. Each of the above types of AuNPs has its own unique properties and advantages for different applications, such as ODFNs, metal nanoclusters, UCNPs, magnetic nanoparticles, silica/polymeric systems, and SERS platforms.

In addition to the molecular fingerprinting, the Raman spectroscopy, especially the SERS, provides an additional dimension for the nano-enabled viral diagnostics by providing a signal enhancement that is mediated by nanoparticles. Gold- and silver-based SERS substrates, magnetic–SERS systems, and Raman nanotags have proven to be useful for sensitive viral detection; and machine learning can help for rapid, multiplexed analysis. However, there are several impediments to clinical translation, including substrate reproducibility, biological matrix effects, spectral standardization and model generalizability.

MXenes are a novel nanomaterial platform with two-dimensional structures that can be used for the detection and study of viruses. They have a high surface area, electrical conductivity and tunable surface functional groups that facilitate the immobilization of viral recognition elements and the construction of electrochemical and optical biosensors. Some MXenes have also been shown to have direct antiviral effects in early studies, which remain limited and depend on MXene composition and experimental conditions. Many more studies are needed to expand the biomedical use of these materials, with a particular focus on oxidation, surface stability, toxicity, reproducibility and validation in physiologically relevant models.

The broad activity of AgNPs is ideal for antiviral applications, while functionalized nanocarriers like AuNPs, metal oxides, or LNPs offer targeted benefits for specific applications. LNPs are especially interesting for delivering nucleic acids, but would need to be further investigated for tissue targeting, repeated dose tolerability, manufacturing consistency and characterisation for regulatory approval. In the same way, virus-templated nanomaterial synthesis also provides structural precision and biomimetic functionality, but faces biosafety, immunogenicity, reproducibility, and scale up issues.

Research should move beyond proof-of-concept demonstrations to standardised, comparative and translational evaluations. Biodistribution, degradation and clearance, immunological effects, toxicity, environmental effects and long-term safety are all important considerations for nanomaterials, along with efficacy. Diagnostic platforms should focus on substrates that can be reproduced, analytical validation of the test, and good performance at the clinical level. Integration with CRISPR-based detection, hybrid nanomaterials, microfluidics, multiplexed sensing, portable Raman systems, and artificial intelligence may further propel the rapid and decentralized viral diagnostics.

Finally, the balance between nanoscale performance and biological safety, reproducibility, scalable manufacturing, regulatory acceptability, cost and clinical utility will be critical to the success of a translation. The future of the field of virology enabled by nanomaterials is not just in the creation of more and more sophisticated nanomaterials, but in the creation of strong and stable platforms that are able to reliably bring the innovations developed in the laboratory to the clinic and technology.

## Glossary

AgNCs: Silver nanoclusters
AgNPs: Silver nanoparticles
AI: Artificial intelligence
AuNCs: Gold nanoclusters
AuNPs: Gold nanoparticles
CRISPR: Clustered regularly interspaced short palindromic repeats
DNA: Deoxyribonucleic acid
ECL: Electrochemiluminescence
ELISA: Enzyme-linked immunosorbent assay
FET: Field-effect transistor
HBV: Hepatitis B virus
HIV: Human immunodeficiency virus
HPV: Human papillomavirus
IAV: Influenza A virus
LAMP: Loop-mediated isothermal amplification
LFIA: Lateral flow immunoassay
LNPs: Lipid nanoparticles
LOD: Limit of detection
ML: Machine learning
mRNA: Messenger RNA
NIR: Near-infrared
NP(s): Nanoparticle(s)
POC: Point-of-care
qPCR: Quantitative polymerase chain reaction
RBD: Receptor-binding domain
RNA: Ribonucleic acid
RPA: Recombinase polymerase amplification
RSD: Relative standard deviation
RT-PCR: Reverse transcription polymerase chain reaction
SARS-CoV-2: Severe acute respiratory syndrome coronavirus 2
SERS: Surface-enhanced Raman spectroscopy
SPR: Surface plasmon resonance
UCNPs: Upconversion nanoparticles
VLPs: Virus-like particles
